# Modulating cyclic nucleotides pathways by bioactive compounds in combatting anxiety and depression disorders

**DOI:** 10.1007/s11033-023-08650-8

**Published:** 2023-07-24

**Authors:** Citlaly Gutiérrez-Rodelo, Shirlley Elizabeth Martínez-Tolibia, Guadalupe Elide Morales-Figueroa, Josué Arturo Velázquez-Moyado, J Alberto Olivares-Reyes, Andrés Navarrete-Castro

**Affiliations:** 1 Department of Pharmacy, Faculty of Chemistry, National Autonomous University of Mexico (UNAM), Mexico City, ZIP 04510 Mexico; 2 Research Center in Applied Biotechnology of the National Polytechnic Institute, Tlaxcala, ZIP 90700 México; 3 Department of Physiology, Biophysics, and Neurosciences of the Center for Research, Advanced Studies of the National Polytechnic Institute (CINVESTAV-IPN), Mexico City, ZIP 07360 Mexico; 4 Department of Biochemistry, Center for Research and Advanced Studies of the National Polytechnic Institute (CINVESTAV-IPN) Mexico City, Mexico City, ZIP 07360 Mexico

**Keywords:** Plant bioactive, Mushrooms bioactive, Second messengers, Signal pathways, Anxiety, Depression, Cyclic nucleotide

## Abstract

Anxiety and depression disorders are highly prevalent neurological disorders (NDs) that impact up to one in three individuals during their lifetime. Addressing these disorders requires reducing their frequency and impact, understanding molecular causes, implementing prevention strategies, and improving treatments. Cyclic nucleotide monophosphates (cNMPs) like cyclic adenosine monophosphate (cAMP), cyclic guanosine monophosphate (cGMP), cyclic uridine monophosphate (cUMP), and cyclic cytidine monophosphate (cCMP) regulate the transcription of genes involved in neurotransmitters and neurological functions. Evidence suggests that cNMP pathways, including cAMP/cGMP, cAMP response element binding protein (CREB), and Protein kinase A (PKA), play a role in the physiopathology of anxiety and depression disorders. Plant and mushroom-based compounds have been used in traditional and modern medicine due to their beneficial properties. Bioactive compound metabolism can activate key pathways and yield pharmacological outcomes. This review focuses on the molecular mechanisms of bioactive compounds from plants and mushrooms in modulating cNMP pathways. Understanding these processes will support current treatments and aid in the development of novel approaches to reduce the prevalence of anxiety and depression disorders, contributing to improved outcomes and the prevention of associated complications.

**Graphical abstract:**

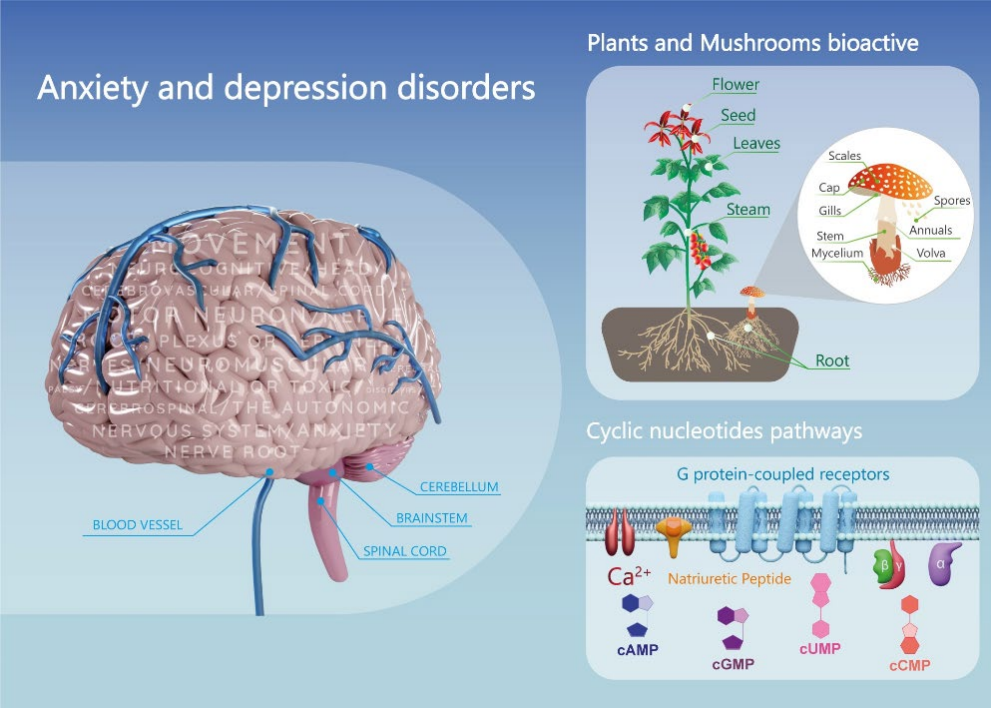

## Introduction

The primary function of the central nervous system (CNS) is to regulate behavior and modulate anatomical and physiological processes through intercellular signaling. Nervous impulse transmission relies on intricate signal transduction cascades within neurons, facilitated by synaptic contacts between cells [1]. To ensure successful information transmission, a multitude of controlling processes involving ion channels, pumps, receptors (neurotransmitters, growth factors, and hormones), enzymes, and structural proteins are essential within the neuronal structure [2]. Specific signals are responsible for eliciting cellular responses, initiating intercellular communication through molecules known as first messengers. These first messengers can act as neurotransmitters, neuromodulators, or both, depending on the binding site of their receptors. Subsequently, they induce the formation of intracellular intermediates called second messengers, which can activate various enzymes, including kinases and phosphatases. Through posttranslational mechanisms, these second messengers modulate numerous functions [3]. Although second messengers may share similar pathways, their regulation, localization, and timing for each cellular response differ, enabling control over diverse effectors and physiological processes. In the CNS, there exist several well-established signal pathways, one of which involves guanine nucleotide-binding protein (G protein)-coupled receptors (GPCRs) that activate enzyme effectors to generate second messengers [2]. Activation of second messengers can also regulate gene expression through posttranslational mechanisms and have long-lasting effects, such as in long-term memory. The second messengers exhibit extensive diversity, encompassing soluble molecules (e.g., diacylglycerol (DAG)), lipids (e.g., phosphatidylinositol 3,4,5-trisphosphate (PIP_3_)), ions (e.g., calcium ion (Ca^2+^), iron (Fe^2+/3+^)), gases (e.g., oxygen (O_2_), nitric oxide (NO)), and free radicals (e.g., hydroxyl radical (• OH)) [1]. Furthermore, cyclic nucleotide monophosphates (cNMPs) are also present, including conventional adenosine 3′,5′-cyclic monophosphate (cAMP), guanosine 3’,5’-cyclic monophosphate (cGMP), and non-conventional uridine 3’,5’-cyclic monophosphate (cUMP) and cytidine 3’,5’-cyclic monophosphate (cCMP) [4].

Regarding the cNMPs, their molecular signaling is already studied with detailed attention in the CNS. For instance, the cAMP signaling is involved in axon growth, plasticity, learning, long-term potentiation, and memory processes. The cAMP is produced from adenosine triphosphate (ATP) by the ATP diphosphate-lyase adenylate cyclase (AC). There are nine AC isoforms (AC1-AC9) integral membrane proteins, and a soluble isoform with different regulatory properties. The cAMP is modulated in response to GPCRs coupled to Gαs (activated) or Gαi (inhibited), triggering the activation of protein kinase A (PKA or cAMP-dependent protein kinase), cyclic nucleotide-gated channels, and the exchange factor directly activated by cAMP (Epac). The cAMP formation promotes the successive activation of the cAMP response element binding protein (CREB). Moreover, CREB controls the transcription of coding genes for neurotransmitter receptors and cyclic nucleotide phosphodiesterase enzymes (PDEs), specialized in the organization of cAMP levels through the degradation of phosphodiester bonds in cAMP molecules and their conversion to ATP[5]. At the same time, learning and memory are controlled by PKA through the activation of cAMP in postsynaptic neurons. This is regulated by N-methyl-D-aspartate (NMDA) receptors, synaptic transmission via aminomethylphosphonic acid (AMPA) receptors, and CREB[6]. PKA also desensitizes G protein-coupled γ-aminobutyric acid (GABA) B receptors involved in regulating AC and cAMP signaling via Gαi. At the same time, AMP-activated protein kinase (AMPK) is activated in response to adenosine nucleotides (AMP, ADP, or ATP). This enzyme is implicated in cellular energy homeostasis, and its role in neuroprotection generates interest[7]. Alternatively, cGMP signaling initiates with guanylyl or guanylate cyclase (GC) through the transformation of guanosine-5’-triphosphate (GTP) to cGMP and pyrophosphate[8]. There are reported two isoforms of the heterodimeric GC (α1β1, α2β1). The first one is found in the cytosol, called nitric oxide (NO)-sensitive GC; it acts as a receptor for NO; the other isoform, a membrane-bound isoenzyme, is activated by natriuretic peptides[9]. Activation of GC is mediated by various factors, including Ca^2+^, NO, natriuretic peptides, and guanylyl cyclase-activating proteins (GCAPs). Subsequently, the generated cyclic guanosine monophosphate (cGMP) activates protein kinase G (PKG), cyclic AMP response element-binding protein (CREB), and cyclic nucleotide-gated channels. cGMP, as a second messenger, plays a crucial role in synaptic transmission and is primarily modulated by Ca^2+^_,_ regulating various functions within the nervous system[10].

**Fig. 1:**
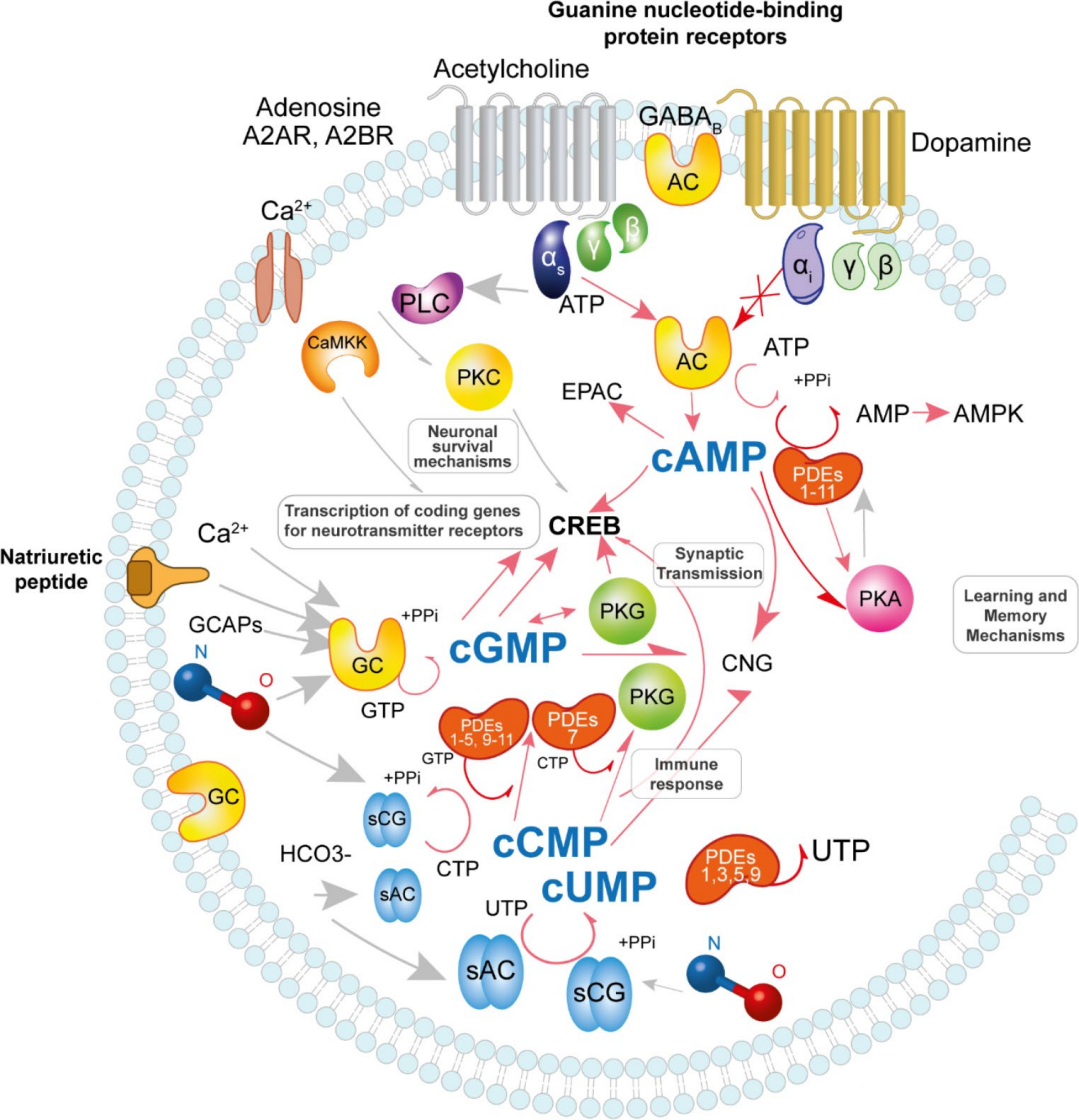
Cyclic nucleotides cross-talk targets. Diverse signal pathways activate enzyme effectors to produce 3′-5′ cyclic adenosine monophosphate (cAMP), 3’,5’ cyclic guanosine monophosphate (cGMP), 3’,5’ cyclic uridine monophosphate (cUMP), 3’,5’ cyclic cytidine monophosphate (cCMP). The cAMP is produced from ATP by the adenylate cyclase (AC) and modulated in response to GPCRs coupled to Gαs (activated) or Gαi (inhibited), triggering the activation of Protein Kinase A (PKA), cyclic nucleotide-gated channels (CNG), and the exchange factor directly activated by cAMP (Epac). The cAMP formation promotes the successive stimulation of cAMP response element binding protein (CREB) controlling the transcription of coding genes for neurotransmitter receptors. PKA regulates the cyclic nucleotide phosphodiesterases enzymes (PDEs) and degrades the phosphodiester bonds in cAMP molecules and their conversion to ATP. AMPK is activated in response to ATP, AMP, and ADP. The cGMP signalling begins with guanylyl or guanylate cyclases (GC) through guanosine-5’-triphosphate (GTP) to cGMP and pyrophosphate. The activation of GC occurs mediated by Ca^2+^, Nitric Oxide (NO), natriuretic peptides, and guanylyl cyclase-activating proteins (GCAPs). The cGMP triggers the activation of protein kinase G (PKG), CREB, and cyclic nucleotide-gated channels. cCMP and cUMP are synthesized by the NO-stimulated guanylyl cyclase (sGC) and the bicarbonate-stimulated soluble adenylyl cyclase (sAC) from cytidine, uridine, adenosine, or guanosine triphosphate

On the other hand, the findings about signaling of cCMP and cUMP non-canonical cyclic nucleotides, had recognized that these cNMPs are synthesized by the soluble NO-stimulated guanylyl cyclase (sGC), and the bicarbonate-stimulated adenylyl cyclase (sAC), from cytidine, uridine, adenosine or guanosine triphosphate[11]. The cCMP and cUMP share the same effectors (PKC, PKG, Hyperpolarization-activated cyclic nucleotide-gated (HCN) channels) and inactivators (PDES) as cAMP and cGMP[12, 13]. The pyrimidine nucleotide signaling and biological functions of cCMP and cUMP in CNS are still under investigation; nevertheless, it has been observed that they may be involved in regulating HCN pacemaker channels and immune responses[13]. As mentioned, one of the key regulators of cNMPs are the PDEs. These enzymes comprise 11 families that degrade and inactivate cNMPs. PDEs exhibit substrate selectivity based on GAF domains or Vmax. Certain PDEs, such as PDEs 1B, 5 A, 6, and 9, specifically target cGMP hydrolysis. Conversely, PDEs 1B, 2 A, 3B, 4B, 7, 8, and 9 prefer cAMP degradation [14]. PDE1, 2,3,5,9,10, and 11 can hydrolyze equally cGMP and cAMP. Nevertheless, since the role of cUMP and cCMP is being appraised, a few studies indicate that PDE7A1 hydrolyzes cCMP, while PDE1B, 3 A, 5 A, and 9 A degrade cUMP[15]. Figure 1 summarizes the targets involved in the crosstalk of cyclic nucleotides.

The concentrations of second messengers are tightly regulated and typically maintained at basal levels ranging from millimolar to nanomolar within cells. However, upon stimulation, their signals are amplified, leading to an increase in their concentration. This releases the messengers and exerts their specific effects on cellular targets. Dysregulation in their production in response to an agonist can result in cellular and organ dysfunction, leading to disease. Emerging evidence suggests that the equilibrium of second messenger levels, such as cAMP, is altered in certain neurological disorders (NDs). For instance, in depression, cAMP signaling is deficient, while IP3/DAG-mediated responses are up-regulated[16]. Moreover, cCMP and cUMP could have relevant functions in brain modulation. It has been reported that cCMP increases the division rate of murine brain cell lines[17], whereas the increase of cUMP in primary astrocyte cells may be involved in developing the pathogenesis of NDs[12].

**Fig. 2:**
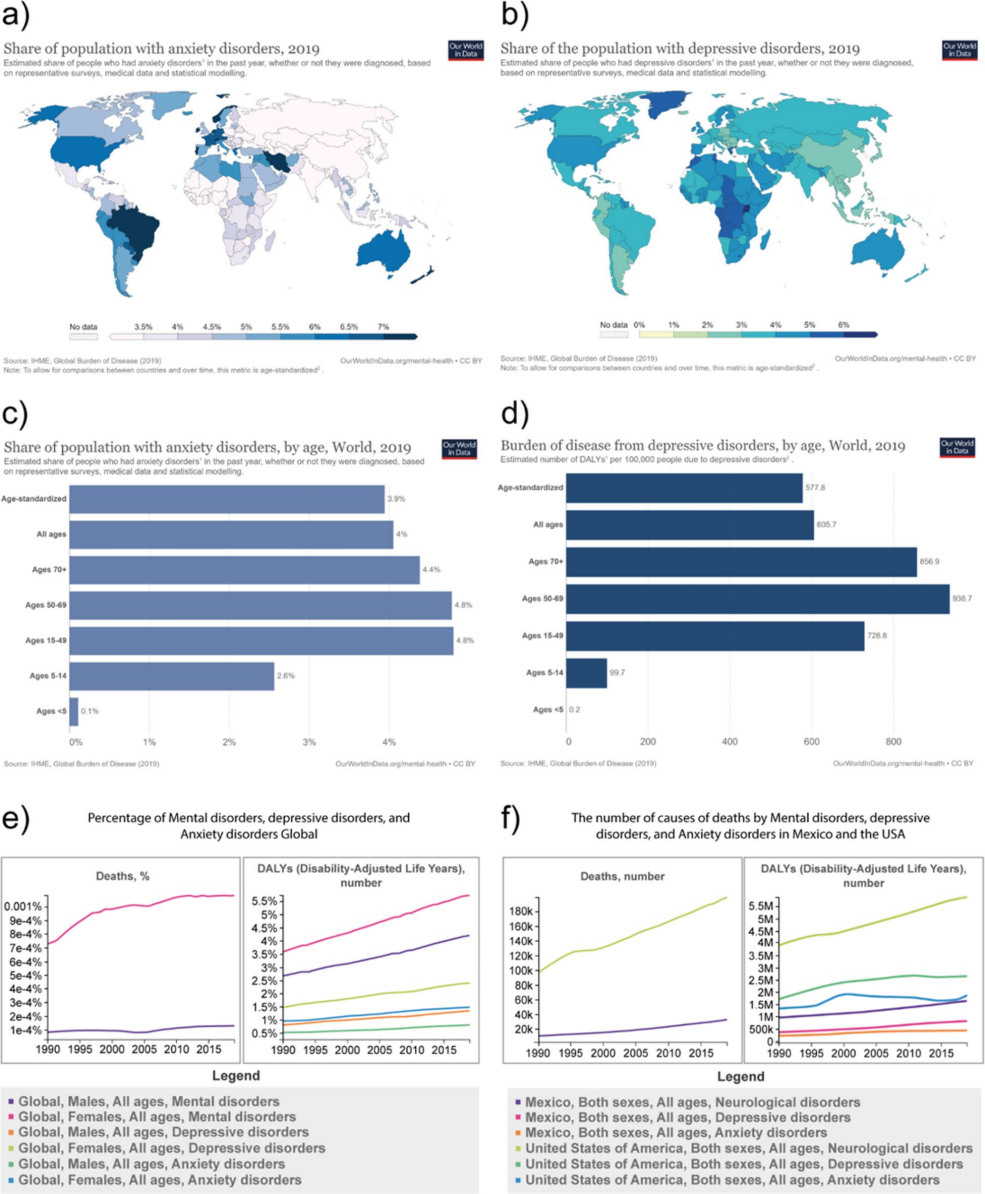
Anxiety and depression population data. **a)** Estimated share of people with anxiety disorders, Global, 2019, based on representative surveys, medical data, and statistical modeling[18]. **b)** Estimated share of people with depressive disorders, Global, 2019, based on representative surveys, medical data, and statistical modeling [18]. **c)** Estimated share of people with anxiety disorders by age, Global, 2019, based on representative surveys, medical data, and statistical modeling[18]. **d)** Estimated burden of depressive disorders, number of Disability-adjusted life years (DAYs), by age, Global, 2019. One DALY represents one year of healthy life[18, 78]. **e**) Percentage of Mental disorders, depressive disorders, and anxiety disorders as causes of death (left) and DAYs (right)[18, 78]. **f)** Number of Mental disorders, depressive disorders, and anxiety disorders in Mexico and USA as causes of deaths (left) and DAYS (right)[18, 78] Copyright © The University of Washington

According to surveys, medical records, and statistics of anxiety and depression disorders, the most common mental health or neurodevelopmental disorder in 2017 was anxiety, with around 284 million persons worldwide diagnosed [18]. More recently, the frequency of anxiety disorders changed from 2.5 to 7% by nation, showing the highest indexes from 1990 to 2019 in Afghanistan (4.7%) (Fig. 2a). In contrast, the share of the population with depression ranges mostly between 2% and 6% worldwide (Fig. 2b)[18]. The Estimated number of DALYs (*Disability-Adjusted Life-Years*) per 100,000 people due to anxiety disorder estimates that people between Ages 50–69 and 15–49 have most lost years in good health (Fig. 2c). The estimations worldwide also suggested that anxiety is prevalent in females (Around 179 million), compared to males (105 million)[18]. In comparison, people 70 years or older have the highest risk of depression(Fig. 2d). In all countries, the median estimate for the prevalence of depression is higher for women than men[19]. Moreover, anxiety prevalence in Mexico has been increasing compared through the years. In 2019, it was reported to be lower (3.5%) than in the SA 2019 (5.7%). In this aspect, 2.9% of persons with anxiety in Mexico were male, while 4.7% were female (Fig. 2e)[19]. Similar findings were observed for depression data in both countries, indicating a tendency to increase over time (Fig. 2e)[19]. In 2020, anxiety and depressive disorders grew pointedly after the COVID-19 disease spreading. The estimations display a 26% and 28% rise correspondingly for anxiety and major depressive disorders in that year[20]. Currently, new endeavors and collaboration to move forward the brain healthiness agenda are compulsory[21, 22].

**Fig. 3:**
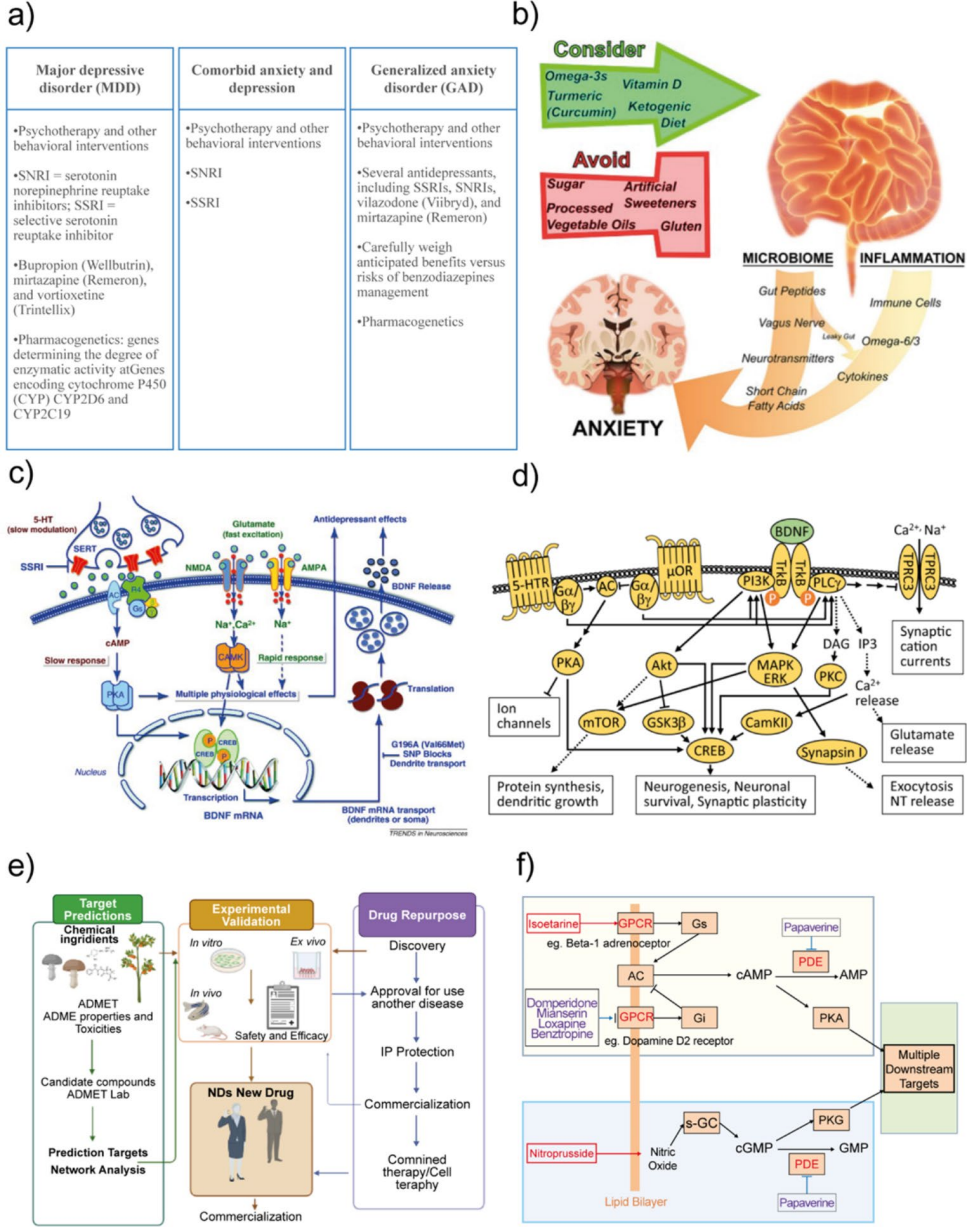
Panorama of pharmacological and molecular approaching for anxiety and depression. **a**) Current pharmacological treatment for major depressive disorder (MDD) and generalized anxiety disorder (GAD) Copyright © 2023 Elsevier Inc[23]. **b**) Regulation of anxiety disorders by Nutrition and affected by microbiome and inflammation. Reproduced with permission from [24], Copyright © 2021 Frontiers. **c**) Signaling pathways regulated by chronic antidepressant treatments. Typical antidepressants, such as 5-hydroxytryptamine (5-HT) selective reuptake inhibitors (SSRIs), block monoamine reuptake by the 5-HT transporter (SERT), triggering to the regulation of postsynaptic GPCRs, which couple to a variety of second messenger systems, including the cAMP–protein kinase A (PKA)–cAMP response element-binding (CREB) pathway[26], Copyright © 2012 CellPress. **d**) Molecular pathways of major depressive disorders. Monoamines affect neurotransmission by modulating intracellular vias acting over diverse proteins such as GPCRs, µOR µ-opioid receptor, TRPC3 transient receptor potential canonical subfamily, TrkB tyrosine receptor kinase B, BDNF brain-derived neurotrophic factor, CREB cAMP responsive element binding protein, DAG diacylglycerol, intercepting downstream processes of neuronal survival and plasticity[25], Copyright © Springer Nature Limited. **e**) Neuroprotective route offered over canonical vias of bioactive with synergistic activity, 10 out of 21 total of these pairs converged on cAMP/PKA, cGMP/ PKG, or both signaling pathways[79]. Copyright © 2022. Published by Elsevier Masson SAS. **f**) A general idea of the NDs drug system development and drug repurposing by the current technology, including systems pharmacology, network analysis, and model systems for experimental validation (*in vitro, ex vivo*, and in vivo), until the commercialization[80]. Copyright © 2021. Published by Elsevier Masson SAS

For anxiety and depression disorders, specialists deal with multifaceted handling; besides pharmacologic care, it also includes a diversity of necessities such as psychotherapy, the addressing of comorbidities, and pharmacogenetics profiles (Fig. 3a) [23]. In addition, nutritional approaches have been described for treating anxiety disorders (Fig. 3b), representing a way to avoid cerebral inflammation[24]. Therapy for these disorders exhibits unsettled pharmacologic prescription by deficient drug intake, nonspecific delivery/distribution, biocompatibility, or side effects. The aspects of the key molecular pathways involved in major depressive disorders, along with the signaling pathways modulated by chronic antidepressant treatments, are elaborated in Fig. 3d [25] and **3c** [26], respectively. Although new drug development is well-described, it involves much effort, high costs, and risks[27]. For instance, the field of Systems Pharmacology offers significant advantages in drug discovery by evaluating natural medicines to identify active molecules and therapeutic targets, thereby providing support for clinical practice [28, 29]. In this regard, network pharmacology-based investigation is starting to be a relevant focus (Fig. 3e). Medicinal plants and their bioactive show neuroprotective or anti-neurodegenerative properties, suggesting a safer alternative against some therapy’s side-effects[30]. These compounds are recognized for safeguarding against inflammatory processes, microbial infections, and oxidative damage[31]. Similarly, there are mushrooms with pharmaceutical assets with a respective content of bioactive secondary metabolites[32]. The exposition to bioactive compounds converges in several processes for increasing, diminishing, or stopping cell responses. Nevertheless, there remains a vast opportunity to explore and harness the potential benefits and mechanisms of action of bioactive compounds derived from plants and mushrooms [33]. Similar to pharmaceutically approved drugs, the administration of bioactive molecules also involves intricate signaling network mechanisms, potentially leading to diverse pathways and the activation of cyclic nucleotide messengers. These interactions can result in significant pharmacodynamic and/or pharmacokinetic effects.The synergistic approach of various compounds, alongside the proposal of small molecules with multiple action targets, must be investigated for more effective clinical treatments for anxiety and depression disorders. For instance, compounds that evoke the activation for the same second messenger over different mechanisms can achieve synergistic neuronal cell defense. For example, the molecule isoetarine activates the pathway β1 adrenergic receptor (β1AR) /Gs/AC stimulating the conversion of ATP to cAMP. At the same time, benztropine antagonizes the M1 muscarinic receptor and blocks the inhibition of AC activity. Thus, both compounds modulate cAMP levels through diverse mechanisms (Fig. 3f)[34].

Cyclic nucleotides play a direct role in anxiety and depression as essential molecules for the optimal functioning of the CNS, influencing neuronal activities. These observations highlight the necessity for enhancing our understanding of the communication within these systems and their involvement in anxiety and depressive disorders. This review aims to highlight the molecular mechanisms through which plant and mushroom-based bioactive regulate cNMPs, thereby influencing the pathologies of anxiety and depression. We will provide an overview of the benefits offered by the bioactive effects in each disease, elucidate the signal pathways associated with their activity, and discuss the corresponding models used for studying these mechanisms.

## Methodology for data collection

The literature search for systematic evidence (original research or review article) was performed using PubMed, Google Scholar, Science Direct, and Scopus electronic databases until May 2023. The main keywords utilized were: “phytochemical,” “plant-derived bioactive for depression or anxiety”; “mushroom-derived bioactive for depression or anxiety”; “cyclic nucleotides and second messengers”; “plants and mushroom bioactive”; “cAMP”; “cGMP”; “cUMP”; “cCMP”; “anxiety disorders”; “anxiety-like disorders”; “depression disorders” among others. The authors manually selected and reviewed all the references included in this study.

### Anxiety disorders pathways

Anxiety and depression are nowadays the foremost individuals and public health burdens and frequently co-morbid mental disorders. Anxiety is an adaptive, defensive condition that supports survival. Its mechanisms are controlled by the prefrontal cortex, associated with limbic cortices, and connected with the amygdala, thalamus, and autonomic structures. Environmental events and genetic predisposition can trigger alterations of these areas in the brain and their neurotransmitter signaling, resulting in the corruption of the activity balance in the emotional centers and the risk of psychopathology. For instance, stress and anxiety are essential to trigger migraine factors or can contribute to the disorder becoming chronic. Compared to the general population or neurological patients, people who suffer from headaches are more likely to endure a co-morbid anxiety disorder[35]. Among anxiety disorders, there are social anxiety, post-traumatic stress disorder, panic, and generalized anxiety disorder, as well as obsessive-compulsive disorder (OCD), separation anxiety disorder (SAD), and phobias[35]. Anxiety is characterized by persistent and excessive fear, extreme nervousness, and feelings of insecurity due to the anticipation of potential threats. If anxiety disorders are not identified and treated, the chronic presence of these threats can significantly increase the risk of harm and mortality in individuals with neurological conditions [36]. Currently, treatments for anxiety disorders are pharmacological and psychotherapeutic interventions. For mood modulation, selective serotonin (5-HT) reuptake inhibitors are prescribed. Benzodiazepines are an effective intervention for acute anxiety, acting via inhibitory neurotransmission by GABA_A_ receptors[35]. Also, Dopamine (DA) neurons participate in anxiety regulation. DA regulates the activity of DA-and cAMP-regulated phosphoprotein (DARPP-32), implicated in the physiological replies of behavior and pharmacological stimuli, including antidepressants[37].

### Depression disorders pathways

On the other hand, according to the quantity and gravity of symptoms, a depressive episode is classified as mild to moderate, major depressive disorder (MDD), severe to severe depression, and bipolar affective disorder[38]. The most common condition is MDD, characterized by persistent changes in mood, faded ability to feel pleasure, loss of energy, disturbed sleep or appetite, and even impaired cognitive function. Depression might involve long-term therapy, and symptoms frequently are severe enough to cause noticeable problems in daily life[39]. Pathological mechanisms involved in depression encompass various factors, such as dysfunction in neurotrophic factors, the hypothalamic-pituitary-adrenal axis (HPA), neuroinflammation, disruption of neurotransmitter systems, stress, metabolic disorders, and the gut-brain axis of the microbiome [40]. In patients with depression, the HPA axis is overexcited under stressful conditions, initiating complications such as hypercortisolemia[41].

Among the neurotransmitter systems participating in depression, DA is a regulator of behavior and precursor to epinephrine and norepinephrine (NE). Additionally, patients with depression have increased DA transport, with presynaptic neurons more effective at reuptake. Further, 5-HT is downregulated, leading to phobias and anxiety. Elevated levels of glutamate have been observed in the blood, cerebrospinal fluid, and brains of patients with depression. Moreover, studies have demonstrated that the inhibition of N-methyl-D-aspartate receptor (NMDAR) function is an antidepressant [40]. Activation of the cAMP/PKA signaling represents an excellent potential for antidepressant effect since the reduction of the cAMP/PKA cascade affects neuronal excitation and synaptic plasticity, triggering depression[42]. Furthermore, the cAMP/ PKA controls the mechanism of depression by regulating the HPA axis, synaptic plasticity, cytokinesis, and transcriptional regulation. For patients that had endured depression, cAMP levels in platelets from the brain were lower than healthy controls. Besides, human post-mortem brain tissue from MDD patients had a lower protein expression of PKA regulatory subunits, indicating that the decrease of the cAMP-PKA pathway has a role in depression progression[43]. Furthermore, antidepressants are associated with typical side effects that can be uncomfortable or potentially trigger other conditions. These side effects may include slight blurring of vision, weight gain, excessive sweating, erectile dysfunction, headaches, arrhythmia, tachycardia, diabetes, a severe reduction in sodium levels, psychosis, decreased appetite, and the emergence of suicidal thoughts[44]. Besides, despite differences in the initial causal route for anxiety and depression, the neural circuitry linked to successful treatment largely overlaps. When anxiety and depression exist together, it causes elevated therapy conflicts and complications[37]. Furthermore, chronic unpredictable mild stress (CUMS) has been shown to induce depression-like behaviors. Preclinical studies have shown decreased expression of BDNF, altered synaptic morphology, and reduced neurogenesis in the hippocampus of rats that were exposed to CUMS[45].

### Mechanism of anxiety and depression disorders as a secondary complication

Anxiety and depression may result from a symptom or a reaction to another disease, NDs, environmental factors exposure, or a prescription side effect. In various NDs, microglia activation leads to the production of inflammatory cytokines, neural damage, and triggers the pathophysiology of anxiety and depression disorders[46]. These disorders are linked primarily to posterior circulation stroke, multiple chemical sensitivity in epilepsy, and Parkinson’s[47]. Likewise, there is a high association between depression and anxiety disorders, and autoimmune thyroiditis[48].

Neurotoxicity encompasses anxiety and depression as symptoms. Neurotoxicity is the dysfunction or impairment of the central and peripheral nervous systems caused by various biological, chemical, or physical factors [49], which have a harmful impact through the interference of metabolic processes, neural function, maldevelopment, or damage to the brain[50]. The effects of neurotoxicity might emerge and disappear fast, develop gradually, relapse over months or years, or produce permanent harm, even causing death[51]. Evidence suggests that dysregulation of cyclic nucleotide signaling pathways can contribute to neurotoxicity, leading to neuronal damage and cell death. Increased levels of cAMP and cGMP have been associated with excitotoxicity, oxidative stress, and inflammation, all of which can contribute to neurodegenerative processes. Conversely, deficits in cyclic nucleotide signaling have been linked to impaired neuroplasticity and reduced resilience to stress, critical factors in anxiety and depression disorders. Additionally, neurotoxic effects are commonly associated with mitochondrial damage, neuronal dysfunction, neurogenesis repression, and apoptosis, which can contribute at the same time to the development of NDs[52].

Neurotoxins act mainly by inhibiting the GABA_A_ receptor, channels (sodium, potassium, Cl^−^ and/or Ca^+ 2^), synaptic vesicle releasers, receptors, blood–brain barrier regulators, and toxins with multiple effects[51]. Furthermore, bacterial infections in NCS can cause neurotoxicity by recognizing cell surface receptors or specific intracellular targets, releasing their toxins at the host cell membrane[53]. The most studied toxins alter the Gαs or Gαi subunits, such as cholera and pertussis toxin, generating uncontrolled levels of cAMP, cGMP, cUMP, or cCMP[53]. Pathogens can control the cNMPs levels of host cells as an advantage through the modulation of eukaryotic ACs[54]. The potential significance of cCMP and cUMP levels in eukaryotic cells is still being explored. Nevertheless, research suggests their involvement in cytotoxicity, as gram-negative bacteria produce these molecules to combat bacterial viruses[55]. As previously discussed, strategies focused on restoring neuron function and promoting neuroprotection are crucial for the treatment and prevention of anxiety and depression. These interactions underscore the importance of considering the broader molecular context when studying the effects mediated by cyclic nucleotides. In conclusion, the study of cNMPs has yielded significant findings regarding the mechanisms implicated in anxiety, and depression disorders. Further exploration into the precise molecular targets and downstream signaling pathways influenced them is essential. Such investigations hold the potential to uncover novel therapeutic approaches and enhance our comprehension of the intricate interactions between cyclic nucleotides and neuropsychiatric conditions.

### Plant-based bioactive in combating anxiety and depression

Plant-based bioactive has shown promise in providing natural alternatives for managing symptoms of anxiety and depression. Here are a few examples: St. John’s Wort (*Hypericum perforatum*) contains bioactive compounds with potential antidepressant properties, such as hypericin and hyperforin, which could increase serotonin, dopamine, and norepinephrine levels. Lavender (*Lavandula angustifolia*) is widely recognized for the calming and soothing effects of its essential oil or lavender-based products due to linalool and linalyl acetate. Passion flower (*Passiflora incarnata*) is known for its sedative and anxiolytic effects. It contains bioactive compounds like flavonoids and alkaloids, which interact with the GABA receptors, promoting relaxation and reducing anxiety. And finally, *Rhodiola rosea* has been traditionally used to combat stress and improve mood because rosavin and salidroside modulate neurotransmitters and reduce symptoms of depression and anxiety[56]. Among plants’ extensively studied bioactive constituents are antioxidant particles such as polyphenols, stilbenes, anthocyanins, flavonoids, lignans, and phenolic acids. Also, carotenoids, including carotenes and xanthophylls, are prominent in this regard.

However, the direct influence of plant-based bioactive on cyclic nucleotides pathways for anxiety and depression can be found reported. For instance, Kuribara et al. observed anxiolytic effects of the *Ginkgo biloba* extract Ginkgolide A. (GA, BN52020) at 1–2 mg/kg dose by increased CREB levels in the dorsal hippocampus and amygdala[57]. Also, in African traditional medicine, the plants *Agapanthus campanulatus F.M. Leighton (Alliaceae), Boophone distica (L.f.) (Amaryllidaceae), Mondia whitei (Hook.f.) Skeels (Asclepiadeace)*, and *Xysmalobium undulatum (L.) Aiton. f. (Asclepiadeace)* has been used to treat depression[58]. *Cervus elaphus L.* (CEL), *Angelica gigas Nakai (AGN), Astragalus membranaceus Bunge (AMB)*, or their mixture (CAA) have been used against fatigue as a secondary side effect of anxiety and depression disorders[59]. AGN and CAA extract treatment increased the activated-AMPK expression, a critical factor in improving muscle injury[59] *Centella asiática* extract (400 and 800 mg/kg) reduced the anxiety, amnesic and depression-like behaviors in a CUMS-induced depression[45].

Likewise, several bioactive have shown remarkable effects for retrieving causes of anxiety, such as neurotoxicity. For instance, the restoration of oxidative status, AChE activities, and increased brain-derived neurotrophic factor (BDNF) were detected in the case of *Portulaca oleracea* seeds extract against acrylamide neurotoxicity[60]. Similarly, the dopaminergic and normal cAMP levels were recovered by treating *Bacopa monnieri* extracts in neonatal rats with neurological dysfunction caused by hypoglycemia[61]. The same effect in restoring cAMP/PKA levels and CREB phosphorylation was observed by curcumin in the cerebral cortex of rats damaged by corticosteroids[41]. Also, *Syzygium aromaticum*[62], *Searsia chirindensis*[63] and *Trichilia catigua* [64] extracts have ameliorated detrimental effects from neurotoxic agents. However, when research is conducted on bioactive compounds with pharmaceutical potential, it is important to consider the possibility of side effects. A notable example is *Prestonia amazonica*, which has exhibited promising neurotransmitter activity for anxiety treatment but has also been associated with adverse cardiovascular outcomes [52].

The plant-based bioactive mainly reported as potential candidates for developing anti-anxiolytics and antidepressants are secondary metabolites of the families, flavonoids, glycosides, alkaloids, terpenes, saponins, and pure compounds such as curcumin, berberine, ginsenosides, and naringenin[65]. In this regard, the evidence demonstrates a wide range of mechanisms for intervention against these disorders. However, the majority of investigations focused on the modulation of cNMP levels in anxiety and depression are aimed at restoring normal neuronal function as shown on Table 1 [36, 45, 56–70].

**Table 1:** Bioactive combating anxiety and depression disorders

Bioactive	Cyclic nucleotide	Plant or Mushroom	Effects	Application Disease	Signal transduction pathway	Models used	Reference
Deoxynivalenol (DON) mycotoxin	cAMP	*Fusarium spp.* mushroom	Decrease in cAMP levels and G1 phase arrest in the cell cycle	Neuroinflammation and cell apoptosis	cAMP/PKA and MAPK pathway	Mouse astrocyte and microglia cell lines	[69]
Caffeine (1,3,7-trimethylxanthine),	cAMP	The genus *Coffea* plant	Behavioral network and cognitive function concerning neuronal apoptosis	Neuroinflammation and cell apoptosis	cAMP-PKA- cAMP response element binding protein (CREB) pathway	Male Sprague-Dawley rats	[81]
Curcumin (1,7-bis(4-hydroxy-3-methoxyphenyl)-1,6-heptadiene-3,5-dione)	cAMP	*Curcuma longa* plant	Increase levels of cAMP, PKA activity and CREB phosphory-lation	Neuroprotective effects	5-HT receptor-cAMP-PKA-CREB signal pathway	Cerebral cortex from rats	[41]
Bacoside A	cAMP	*Bacopa monnieri* plant	The dopaminergic and cAMP imbalance	Neurological dysfunction from hypoglycemia	cAMP-PKA signal pathway	Wistar neonatal rats	[61]
*Centella asiatica* extract	cAMP	*Centella asiática* plant	Reversed the anxiety and depression	Anxiety and depression	Brain-derived neurotrophic factor (BDNF), cAMP response element binding protein (CREB)	Chronic unpredictable mild stress-induced rats	[45]
Concentrated ethanol extract from the leaves	cAMP and/or cGMP	*Michelia champaca L. (Magnoliaceae)* plant	Antidepressant and anxiolytic properties	Anxiety and depression	Dopamine-mediated signal transduction and Human potassium channel KCSA-FAB and human 5-HT transporter	Swiss albino mic and *in silico*	[37]
Ferulic acid (FA, 4-hydroxy-3-methoxycinnamic acid)	cAMP	Naturally present in several plants and foods	Antidepressant-like effect	Depression	PKA/ CAMKII Calcium/calmodulin-dependent protein kinase II (CaMKII) / PKC/MAPK/PI3K	Mice	[82]
Curcumin (1,7-bis(4-hydroxy-3-methoxyphenyl)-1,6-heptadiene-3,5-dione)	cAMP and/or cGMP	*Curcuma longa L* plant	Alleviate Anxiety-like behaviors The microbiota-gut brain communications	Anxiety	Increased phosphatidylcholine in the prefrontal cortex	Dextran sulfate sodium salt (DSS) in mice	[83]
Ginkgolide A	cAMP	*Ginkgo biloba* plant extract	Anxiolytic effects	Anxiety	Increased CREB levels in the dorsal hippocampus and amygdala regions	Rats	[57]
Cannabidiol	cAMP	*Cannabis * *sp* plant	Prevent anxiolytic- and depressive-related behavior in early-life	Anxiety	Long-lasting molecular changes in mTOR	Female mice	[84]
Flavonoids	cAMP	*Agapanthus campanulatus (AC), Boophone distica (BD), Mondia whitei (MW)* and *Xysmalobium undulatum (XU)* plants	Antidepressant activity	Depression	Serotonin, Dopamine transporter	Male Wistar rats, male albino Swiss mice and male C57BL/6J mice	[58]
Saponins of methanolic extract	cAMP and/or cGMP	Roots of *Asparagus racemosus Linn.* plants	Antidepressant activity	Depression	Serotonergic and the noradrenergic systems and augmentation of antioxidant defenses.	Rats	[85]
GABA (g-aminobutyric acid)	cAMP	*Apocynum venetum* plant	Shortened sleep latency	Insomnia Anxiety/ Depression	GABAergic transmission	Humans	[86]
Methanolic extract	cAMP	*Benincasa hispida (Cucurbitaceae)* plant	Antidepressant-like effect	Depression	Dopaminergic, α1-adrenergic, serotoninergic, and GABAergic systems.	Swiss male albino Mice	[87]
Hericenones and erinacines	cAMP	*Hericium erinaceus* mushroom	Reduce depression and anxiety	Anxiety and depression	Nerve growth factor	Females	[88]
Mycelium extract (80%) and fruiting body (20%) extract	cAMP	*Hericium erinaceus* mushroom	Reduced depression and anxiety, and improved sleep disorders	Anxiety and depression	pro-BDNF and in the pro-BDNF/BDNF ratio	overweight or obese participants	[67]
N,N-Dimethyltryptamine (DMT)	cAMP	*Prestonia amazonica (Apocynaceae)* plant	Activation of adenylyl cyclase and cAMP increase	Anxiety and psychosis	Neurotransmitter function Trace amine-associated receptors (TAARs)	HEK293 cells	[89]

Adenosine 3′,5′-cyclic monophosphate (cAMP), Guanosine 3’,5’-cyclic monophosphate (cGMP), protein kinase A (PKA), cAMP response element-binding protein (CREB), Extracellular signal-regulated protein kinases 1 and 2 (ERK1/2), CAMKII Calcium/calmodulin-dependent protein kinase II (CaMKII), Brain-derived neurotrophic factor (BDNF).

### Mushroom-based bioactive boundaries in anxiety and depression disorders

While mushrooms contain various bioactive compounds that may have potential therapeutic effects, the specific mechanisms of action and their impact on cyclic nucleotides in anxiety and depression have not been extensively studied. Certain species of mushrooms, such as those belonging to the *Psilocybe* genus, contain psilocybin and psilocin. These compounds are classified as psychedelics and are known to interact through various mechanisms, including modulating serotonin pathways, and promoting neuroplasticity. Research on psilocybin-assisted therapy has yielded promising results in treating anxiety, depression, and other mental health conditions. This therapeutic approach typically follows a structured framework with trained therapists to ensure safety and optimize the potential benefit [66]. However, the direct influence of Psilocybin-containing mushrooms on cyclic nucleotides for these conditions is not well-documented.

Mushrooms contain various bioactive compounds, including multiple alkaloids, terpenes, and polysaccharides. Some of these compounds have demonstrated potential therapeutic effects, such as anti-inflammatory, antioxidant, and neuroprotective properties. The best examined are polysaccharides, polyphenols, glycoproteins, terpenoids, metal-chelating compounds, peptides, lipids, and oxidative enzymes[32]. Particularly in the search for mushroom-based bioactive against anxiety and depression, *Hericium erinaceus* (*H.E*.) mycelium extract repaired the depleted expression levels of anxiety and depression controlling molecules 5-HT, norepinephrine, and DA in the hippocampus of restraint-stressed animals, possibly by inhibiting the enzymatic degradation of Monoamine Oxidase[67]. The *H.E* extract has been tested in preclinical trials, and researchers are currently recruiting for a future clinical trial with potential outcomes for cognitive functioning[68].

It is important to acknowledge that the effects of mushrooms, like plants, can vary significantly depending on factors such as the species, growth conditions, dosage, set (mindset), and setting (environment). This variability poses challenges in the standardization and quality control of mushroom-based products. Inconsistencies in bioactive content can lead to variations in efficacy and affect the reproducibility of neuroprotective effects. Some bioactive compounds may interact with medications or have side effects in certain populations. In this regard, it has been reported that mushrooms can cause neurotoxic effects producing neuroinflammation and cell apoptosis. Mycotoxins are secondary metabolites synthesized by various mushroom species such as *Fusarium, Aspergillus, Penicillium, Alternaria*, and *Claviceps spp*. Mycotoxins have been shown to induce significant neuronal damage, causing high oxidative stress, increased Reactive oxygen species (ROS) production and apoptosis via MAPK-JNK-c-Jun and ERK pathways. Also, the mycotoxin deoxynivalenol (DON) from *Fusarium sp*, decreases cAMP levels and has neurotoxicological effects[69]. Moreover, in the worst cases, mycotoxins can contribute to the pathogenesis of neurodegenerative diseases such as the Ochratoxin A produced by *Aspergillus ochraceus* and *Penicillium verrucosum*[70]. Intoxication with T-2 toxin from *Fusarium spp* causes lethargy, ataxia, and emesis in humans and animals, by a mechanism that may involve a change in the concentration of neurotransmitters in the brain. Also, the aflatoxins produced by *Aspergillus fumigatus* are considered the most toxic of mycotoxins. AflatoxinB1 inhibits cAMP and cGMP hydrolysis reactions and may modify PDE activity[71].

The summary of findings on bioactive compounds derived from mushrooms for the treatment of anxiety and depression can be found in Table 1.

### Summary challenges and perspectives

As stated, cNMPs play a crucial role in investigating biochemical and physiological signal transduction pathways within the CNS. They serve as amplifiers of signals initiated by neurotransmitters, hormones, and growth factors. Numerous studies have emphasized their involvement in the regulation of mood and behavior. Disruptions in cAMP and cGMP levels have been observed in animal models of anxiety and depression, highlighting their potential relevance in these disorders. Targeting cNMPs pathways has shown therapeutic promise, with pharmacological agents such as PDE2 inhibitors demonstrating efficacy in alleviating symptoms of depression[72, 73].

This review primarily focuses on the contribution of plant and mushroom-derived bioactive and their activity in anxiety and depression-related neurological disorders. It particularly emphasizes the modification of cAMP, cGMP, cCMP, and cUMP pathways as potential treatment and prevention approaches. Bioactive compounds offer neuroprotection and promote neurotrophic factors while also providing anti-inflammatory and antioxidant properties. Furthermore, they can be advantageous in combating neurotoxic disorders by mitigating early neuronal damage and potentially preventing more severe disease outcomes. Additionally, highlights the involvement of certain mushroom toxins that can activate GPCRs signaling and modulate cAMP and cGMP pathways, contributing to neuroinflammation and neurotoxicity as underlying causes of these diseases. Moreover, the role of non-conventional cyclic nucleotides, such as cCMP and cUMP, is mentioned in relation to their ability to control bacterial toxins.

**Fig. 4:**
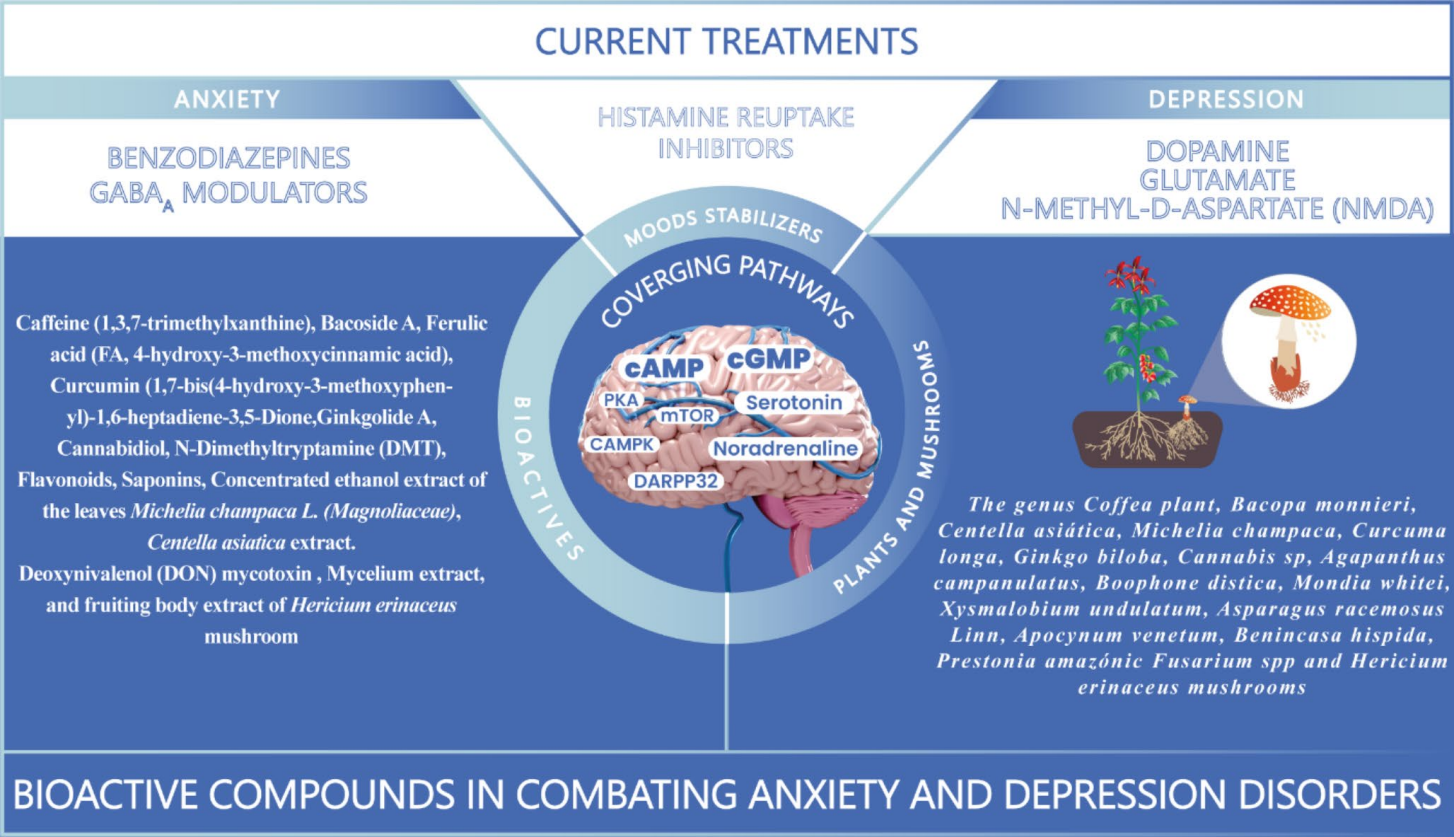
Resume of plants and mushroom-based bioactive and regulated pathways by cNMPs in anxiety and depression disorders

Figure 4 summarizes the findings of bioactive that regulate cNMPs in anxiety and depression disorders. For these disorders, bioactive promotes highlining effects over nerve growth factors expression. In addition, stimulates cAMP/PKA/CREB/mTOR /CaMK/ DARPP-32 pathways and GABA, DA, and 5HT neurotransmitters. A limited amount of information focuses on cGMP/GC/NO signaling. In the context of anxiety and depression disorders associated with neurotoxicity, bioactive compounds primarily modulate GABA_A_, 5-HT, and glutamatergic receptors through the *PKA cAMP* pathway. The anti-anxiety and depression effects were collected from 15 bioactive plants such as *Bacopa monnieri, Centella asiática, Michelia champaca, Curcuma longa, Ginkgo biloba, Cannabis sp, Agapanthus campanulatus, Boophone distica, Mondia whitei, Xysmalobium undulatum, Asparagus racemosus Linn, Apocynum venetum, Benincasa hispida*, and *Prestonia amazonic* and the mushrooms *Fusarium spp* and *Hericium erinaceus*. This information is significant for physiological functions and disease outcomes such as neuroinflammation, cell apoptosis, neurotoxicity, and neuroprotective effects.

**Fig. 5:**
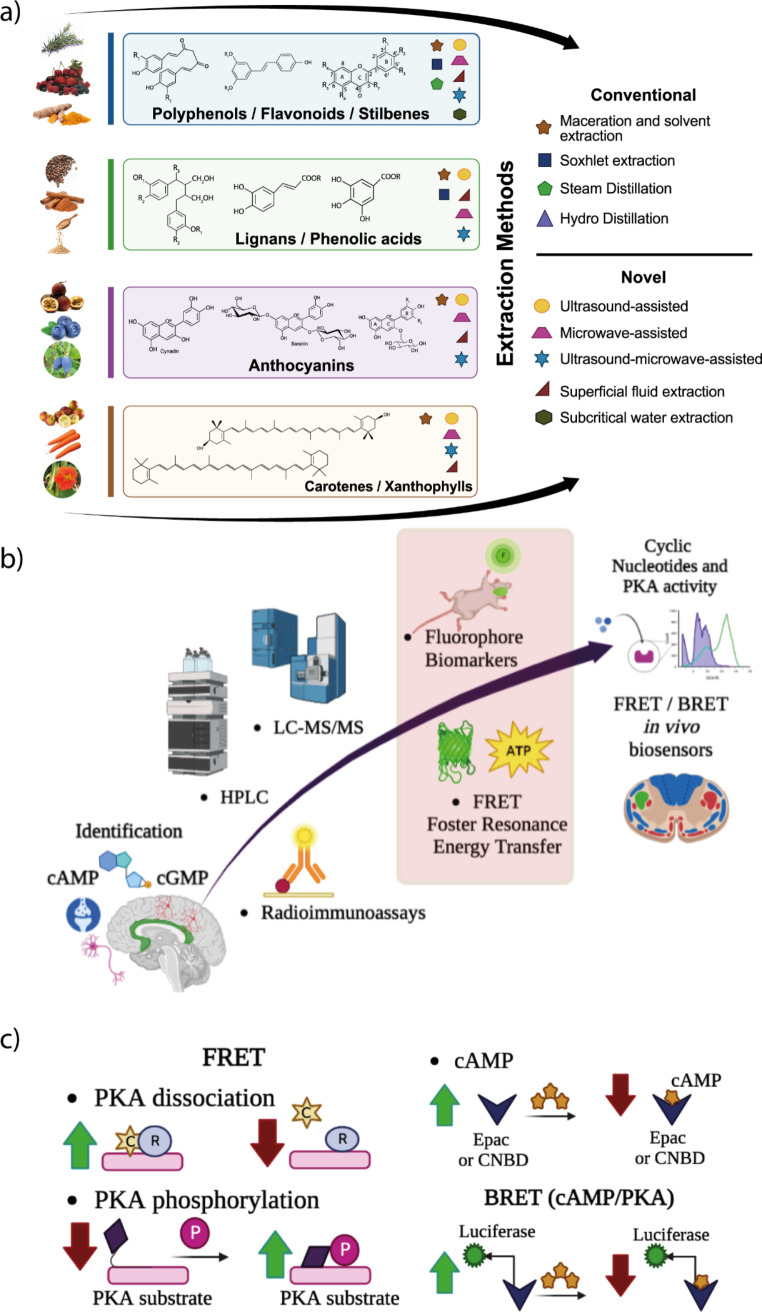
Conventional and novel methods for extracting and purifying the most used bioactive compounds from natural sources with neuroprotective potential. **(a)** Useful techniques for Cyclic Nucleotides detection. **(b)** Schematic view of traditional and current strategies for cAMP and cGMP identification. **(c)** Mechanisms of cAMP and PKA sensors. The FRET technique is based on fluorophores interaction which has been used to report changes in protein conformation or protein-protein interaction. Since PKA holoenzyme has two regulatory subunits, the dissociation (in the presence of cAMP) or phosphorylation can cause variations in the FRET levels (left panel). Another strategy (right panel) is based on the interaction of cAMP with an effector molecule Epac (Exchange proteins activated by cAMP) or CNBD (Cyclic Nucleotide Binding Domain), which undergoes a conformational change and provides FRET signal variations. Similarly, BRET sensors (Bioluminescence-Resonance Energy Transfer) also use cAMP binding and PKA phosphorylation to induce changes in the activity of luciferase. High FRET and low FRET are represented by green and red arrows, respectively. Summarized information is based on the revision of Massengill et al. 2021 [375] *Created with Biorender*

Plant and mushroom-based bioactive hold the potential to contribute to the development of innovative strategies aimed at addressing neurotoxicity and enhancing neurological health due to anti-inflammatory, antioxidant, and neurotransmission-modulating properties. Additionally, advancements in analytical tools, genome mining, and engineering approaches have revitalized the natural bioactive drug development field, paving the way for new possibilities and avenues for exploration[74]. However, challenges such as variability in bioactive composition, limited clinical evidence, and potential toxicity in the case of mushrooms need to be addressed. Further research, including well-designed clinical trials, must unlock their full potential for anxiety and depression. It is worth mentioning that there are technical challenges in isolating plant and mushroom bioactive. In this context, Fig. 5a shows conventional and novel methods for extracting and purifying the most used bioactive compounds from natural sources. Biomolecules extraction included bioactive structures of caffeine (1,3,7-trimethylxanthine), bacoside A(C_41_H_68_O_13_), Ferulic acid (FA, 4-hydroxy-3-methoxycinnamic acid), Curcumin (1,7-bis(4-hydroxy-3-methoxyphenyl)-1,6-heptadiene-3,5-dione, Ginkgolide A (C_20_H_24_O_9_), Cannabidiol (2-[(1R,6R)-6-Isopropenyl-3-methylcyclohex-2-en-1-yl]-5-pentylbenzene-1,3-diol), Flavonoids (derived from 3-phenylchromen-4-one (3-phenyl-1,4-benzopyrone), Saponin (C_58_H_94_O_27_), *N,N*-Dimethyltryptamine (DMT), Deoxynivalenol (DON) mycotoxin and ethanolic extractions from leaves of *Michelia champaca L.* (*Magnoliaceae)* and *Centella asiatica*.

The beneficial impact of CREB modulation was well observed in the gathered data in relation to various bioactive compounds. However, elucidating the subsequent up- and downstream signaling events following bioactive intake/administration and their potential genetic or protein modifications over time has proven to be challenging [75]. For instance, it has been hypothesized that the effects of molecular messengers are highly dependent on the analyzed cell type. Moreover, spatially diverse pools of cNMPS activate localized protein kinases close to their specific targets in each NDs[5]. Regarding to the strategies for cAMP and cGMP identification, valuable techniques for detection are displayed in Fig. 5b. In addition, current practices such as Fluorescence resonance energy transfer (FRET) and nanoscale live-cell scanning ion conductance microscopy (SICM) have facilitated the display of cAMP signals to correlate physiological changes. Therefore, mechanisms of cAMP and PKA sensors are shown (Fig. 5c). In addition, the cAMP and cGMP analogs have recently emerged as attractive opportunities to explore these challenges. For instance, cAMP analogs N(6), O(2)’-dibutyryl-cAMP and 8-chloro-cAMP have been tested in phase II clinical trials as drug candidates[76]. Likewise, live-imaging fluorescence microscopy and more approaches to fluorescence deliver a range of instruments to investigate virtually any cellular process under the microscope[5]. Furthermore, exploring tissue-specific gene modifications can offer new avenues for unraveling the mechanisms of cNMPs at the cellular, organ, or systemic level. It has been demonstrated that each tissue possesses distinct gene expression patterns, thereby establishing cellular dependencies on cNMPs. This approach holds promise for gaining deeper insights into the specific roles and functions of cNMPs in different tissues [77]. However, due to technical difficulties and a lack of specialized techniques, physiological cNMPS and related disease levels are reported solely in *post-mortem* tissues or in vitro studies. Also, records on continuous monitoring of cNMPS levels after bioactive intake/administration over time are yet to be investigated. This evidence may aid in understanding cNMPS behavior as a biomarker and/or for treatment improvement. Thus, new approaches are needed to realize cNMPs compartmentalization nature.

Concerning non-canonical cCMP and cUMP, research studies remain limited. The present review describes the findings of cCMP and cUMP that, until this date, seems to point between the heart and the nervous system cell functions [82]. Nevertheless, their role as molecules modulated by bioactive and their utility in health and anxiety or depression is to be investigated. Additionally, synthesis and degradation mechanisms must be studied in tissues and in vivo models.

The compiled bioactive in the present review can contribute to the findings of potential new pharmacological approaches for anxiety and depression disorders, for instance, by controlling multiple pathways. With a multi-target therapeutic approach, there is a chance to combat one disease from many paths, greatly enhancing the probability of disease adjustment. Also, it can be advised to reduce doses and achieve synergistic therapeutic effects[40]. Furthermore, in the pursuit of effectiveness, bioactive tests for another disorder could be practical for some others, as described, due to the signal pathways that can be shared. Overall, since some PDE inhibitors and AC or GC modulators are currently in preclinical, clinical trials or even commercially available, coupled with extensive information on regulatory mechanisms, new detection, and isolation techniques, there is a motivation for exploring bioactive that modulate cNMPs signaling pathways as prodigious candidates and the closest targets for supporting anxiety and depression disorders treatment.

## Data Availability

Not applicable.
